# Evaluating complex interventions in context: systematic, meta-narrative review of case study approaches

**DOI:** 10.1186/s12874-021-01418-3

**Published:** 2021-10-25

**Authors:** Sara Paparini, Chrysanthi Papoutsi, Jamie Murdoch, Judith Green, Mark Petticrew, Trisha Greenhalgh, Sara E. Shaw

**Affiliations:** 1 Nuffield Department of Primary Care Health Sciences, University of Oxford, Radcliffe Observatory Quarter, Oxford, OX2 6GG UK; 2 School of Population Health & Environmental Sciences, King’s College London, London, UK; 3 Wellcome Centre for Cultures & Environments of Health, University of Exeter, Exeter, UK; 4 Public Health, Environments & Society, London School of Hygiene & Tropical Medicine, London, UK

**Keywords:** Complex interventions, Evaluation, Case study methods, Context, Qualitative research, Mixed methods research, Literature review, Meta-narrative

## Abstract

**Background:**

There is a growing need for methods that acknowledge and successfully capture the dynamic interaction between context and implementation of complex interventions. Case study research has the potential to provide such understanding, enabling in-depth investigation of the particularities of phenomena. However, there is limited guidance on how and when to best use different case study research approaches when evaluating complex interventions. This study aimed to review and synthesise the literature on case study research across relevant disciplines, and determine relevance to the study of contextual influences on complex interventions in health systems and public health research.

**Methods:**

Systematic meta-narrative review of the literature comprising (i) a scoping review of seminal texts (*n* = 60) on case study methodology and on context, complexity and interventions, (ii) detailed review of empirical literature on case study, context and complex interventions (*n* = 71), and (iii) identifying and reviewing ‘hybrid papers’ (*n* = 8) focused on the merits and challenges of case study in the evaluation of complex interventions.

**Results:**

We identified four broad (and to some extent overlapping) research traditions, all using case study in a slightly different way and with different goals: 1) developing and testing complex interventions in healthcare; 2) analysing change in organisations; 3) undertaking realist evaluations; 4) studying complex change naturalistically. Each tradition conceptualised context differently—respectively as the backdrop to, or factors impacting on, the intervention; sets of interacting conditions and relationships; circumstances triggering intervention mechanisms; and socially structured practices. Overall, these traditions drew on a small number of case study methodologists and disciplines. Few studies problematised the nature and boundaries of ‘the case’ and ‘context’ or considered the implications of such conceptualisations for methods and knowledge production.

**Conclusions:**

Case study research on complex interventions in healthcare draws on a number of different research traditions, each with different epistemological and methodological preferences. The approach used and consequences for knowledge produced often remains implicit. This has implications for how researchers, practitioners and decision makers understand, implement and evaluate complex interventions in different settings. Deeper engagement with case study research as a methodology is strongly recommended.

**Supplementary Information:**

The online version contains supplementary material available at 10.1186/s12874-021-01418-3.

## Background

There is growing interest in methodological approaches that support meaningful evaluation of complex interventions in health care [1–3], offer to address issues of causality in complex systems [4, 5] and grapple with the thorny issue of what counts as ‘context’ and what as ‘intervention’. Case study research focuses on in-depth explorations of complex phenomena in their natural, or real-life, settings [6], enabling dynamic understanding of complexity, and surfacing the different logics underpinning causal inferences. While there is wide variation in case study research and its implementation, this approach can provide vital evidence for those concerned with internal and external validity and the likely effects of complex interventions across different settings. However, there is currently limited information about how the diversity of available case study research approaches can support implementation and evaluation of complex interventions in health care [7].

To address a recognised lack of clarity on how researchers should conduct and report empirical case studies [7], and especially to address the knotty problem of how context should be understood and operationalised in such studies [6], we undertook a systematic meta-narrative literature review. This was part of the Triple C (Case study, Context and Complex interventions) study that aims to develop guidance and standards for reporting case study research into the influence of context in complex health interventions. We begin by summarising approaches used in evaluating complex interventions, and setting out the principles and methods of meta-narrative review. We then present four research traditions, each comprising a meta-narrative (that is, an unfolding story of empirical research and the underpinning assumptions and theory), relating to case study research on context and complex interventions, arguing that those involved in intervention evaluation need to make explicit and transparent choices about the type/s of case study on which their research draws. Doing so will increase understanding of the knowledge produced and potential for transferability of findings.

### Approaches to understanding and evaluating complex interventions

The current interest in case study research represents a shift away from studies of complex interventions that involve a standardised sequence of developing a structured, multi-component intervention, testing it in a RCT [8] and following a somewhat prescriptive approach to implementation. This well-established approach conceptualised complexity as residing in interventions that consisted of multiple components acting independently and inter-dependently, making it difficult to identify the ‘active ingredient’ [9] leading to intervention effects. In the UK, this approach formed the basis of the Medical Research Council’s (MRC) 2000 framework for the development and testing of complex interventions [9] and, later, guidance on conducting process evaluations [10].

Ways of conceptualising, developing, implementing and evaluating complex interventions have since shifted significantly, in terms of where the complexity is assumed to lie (from the intervention to the system to the interaction between the two [11, 12]), and how best to study it (from the RCT to a more pluralistic approach that gives appropriate methodological weight to real-world case studies [4, 6, 13]). In public health and health services research, it is now widely accepted that evaluating complex interventions requires a wide range of evaluative evidence, particularly where RCTs and quasi-experimental studies are either not feasible or inappropriate. Many of the critiques of established research designs are linked to the challenge of ‘context’, which is crucial to understanding intervention effects in particular settings [14] but often brings ‘noise’ and uncertainty and so is often controlled for and excluded a priori.

Evaluation frameworks and guidance have adapted to account for the necessary behavioural change and organisational involvement required to implement the intervention, the level of variability of outcomes and the degree of intervention adaptability needed, the importance of non-linearity and iterative local tailoring, and the need to pay attention to the social, political or geographical context within which interventions take place [10, 15, 16]. Recently, the MRC and National Institute for Health Research (NIHR) commissioned an update of guidance on complex interventions [17]. Much uncertainty remains about the best methods for evaluating and implementing complex interventions. For instance, there is a need for better designs that can address questions of causation in natural experiments and questions of complex causation [18]. This includes more consideration of the potential of non-experimental, mixed methods and process-based approaches, appreciation of the different logics of causality, and use of case study research to understand context [13, 19–21].

Case study research is sometimes regarded as providing ‘poor evidence’ for causality [7, 22]. But empirical case studies can enable dynamic understanding of complex challenges, help strengthen causal inferences (particularly when pathways between intervention and effects are non-linear) and provide evidence about the necessary conditions for intervention implementation and effects [23, 24]. This is because they ‘*generally address multiple variables in numerous real-life contexts, often where there is no clear, single set of outcomes*’ ([25] p775), making case study an important methodology for studying complexity and an invaluable resource for understanding the influence of context on complex system-level interventions.

There are many ways to conceive and operationalise context [26]. An influential definition from the MRC guidance refers to context as ‘*anything external to the intervention which impedes or strengthens its effects*’ ([10] p2). This intervention-centred approach reflects concerns (e.g. of researchers, funders) to prepare the grounds for an intervention, plan implementation and assess transferability across settings. Another approach sees context as relational and dynamic, and as emerging over time in multiple different levels of the wider system [27]. Rather than an external environment into which an intervention is introduced, context is seen as the numerous opportunities, constraints, issues and happenings that become salient as the intervention unfolds. In the latter view, context cannot be conceptualised and ‘measured’ separately from the intervention.

Most health-related interventions happen in complex systems made up of multiple evolving interactions [4]. As complex interventions typically depend on elements of context for their effectiveness and there is limited control over such context (it cannot be measured or isolated), challenges arise for a priori hypotheses, evaluation and translation beyond a specific study setting [28, 29]. Case study research offers a much-needed resource for understanding the evolving influence of context and for enabling users to know what the likely effects of complex programmes or interventions will be in those settings [30–34].

## Methods

### Objectives and focus of the review

The Triple C study was funded via a commissioned call from the UK MRC Better Methods, Better Research panel, focused on improving the quality of case study research into the influences of context on complex system-level interventions. Research questions were as follows:

- Which research (or epistemic) traditions have considered case study research, and how does each conceptualise and operationalise case study and context?
- What theoretical and methodological assumptions underpin these research traditions?
- What insights can be drawn about the use of case study research to understand context by combining and comparing findings from studies coming from different traditions?
- What are the main methodological insights and/or empirical findings, particularly in relation to context, and the relationship between context and intervention in health research?
- How do these findings relate to how case study research has been used in studies of complex health interventions? What, if anything, is missing?

The work reported here aimed to: (i) review and synthesise the literature on case study research methods across relevant disciplines, and (ii) determine relevance to the study of contextual influences on complex interventions in health systems and public health research. A subsequent phase involves development and testing of guidance and publication standards using a Delphi panel, workshop, and pilot testing on real-world case studies.

### Methodological approach

We conducted a meta-narrative review [35, 36]. Originally developed by Greenhalgh and colleagues to explain the disparate data encountered in their review of diffusion of innovation in health care organisations [32], the meta-narrative review process is guided by six principles (Table 1) and involves looking beyond the content of literature to the way it is framed.

**Table 1:** Six principles guiding meta-narrative review^a^

Principle	Definition	How we addressed this in the study
Pragmatism	Be guided by what will be most useful to the intended audience	Explicit orientation to MRC stated focus to develop guidance on how to study complex interventions and context using case study methodology
Pluralism	Illuminate the topic from multiple angles	Wide inclusion criteria intended to capture all relevant studies that can be broadly defined as ‘case study’
Historicity	Capture how research traditions have unfolded over time	Consider how later studies drew on, and built on, earlier studies within a tradition, with particular focus on ‘seminal’ (well-regarded, highly-cited) early papers in each tradition
Contestation	Examine ‘conflicting’ data across traditions to generate higher-order insights	Identification and exploration of higher order ‘narrative threads’ (e.g. about what a case study is) being exchanged, contrasting or bridging across the different traditions
Reflexivity	Continually reflect on emerging findings as the review progresses	Regular meetings between team members to share findings and discuss interpretations, including reflecting on how best to produce a useful set of guidance
Peer-review	Present emerging findings to an external audience and use their feedback to guide further reflection and analysis	Delphi panel (currently ongoing) where the findings of this review are presented to a panel of 35 scholars and practitioners for individual scoring, free-text feedback and structured discussion; conference presentations; pilot testing of guidance and meta-narrative review with researchers who have published case studies

^a^ Adapted from Wong et al. [37]

### Search strategy and selection of documents

**Fig. 1:**
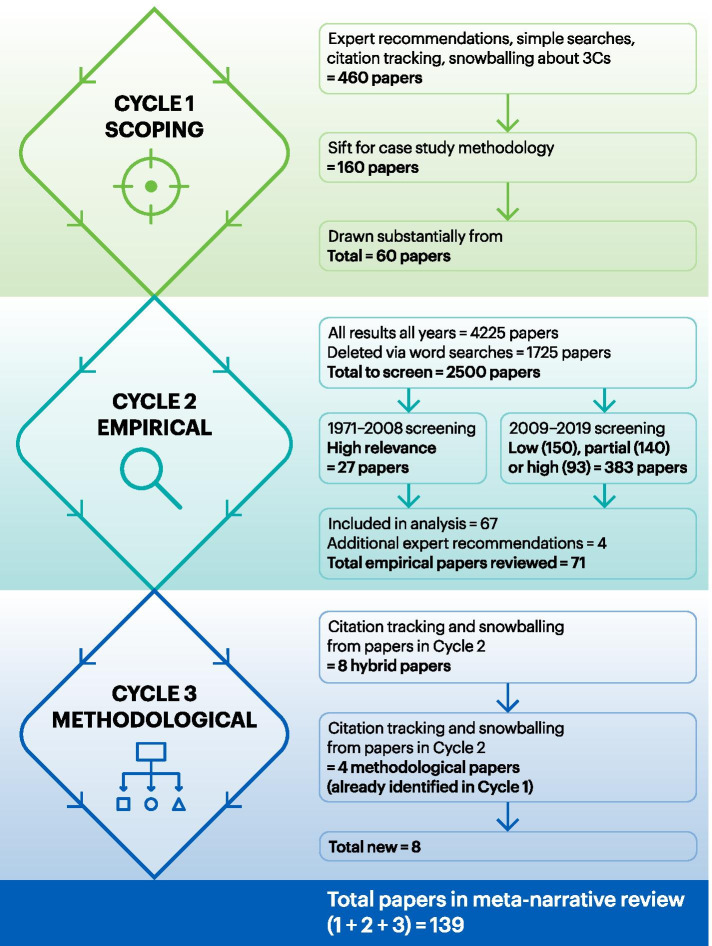
Overview of search results across cycles

Our review was carried out in three linked cycles (Fig. 1). Cycle 1 comprised a scoping review of seminal texts on case study methodology and on context, complexity and interventions. In addition to sources known to the research team, we located papers through database searches, expert recommendations (e.g. via social media), citation tracking and snowballing. This informed our detailed search strategy in Cycle 2, developed with an information specialist and using multiple search terms to capture the empirical literature in which *case study, context* and *complex interventions* overlapped (see Additional file 1: Appendix 1). We searched 11 databases: Medline, Embase, PsycINFO, CAB Abstracts, Science Citation Index, Social Sciences Citation Index and Arts & Humanities Citation Index, ERIC, CINAHL, ASSIA, Sociological Abstracts and PAIS Index. We searched the databases from November 2019 back to when their respective records began (the earliest record returned was from 1971). After removal of duplicates, results were imported into Endnote for screening and classification. A sample of 50 papers were screened by SP, CP and SS and discussed in team meetings to progressively refine screening criteria and develop consensus within the team. SP captured the reasoning behind inclusion and exclusion decisions. SP independently screened and sorted all records from 2009 to 2019, initially by reviewing titles and abstracts and then by reviewing full papers in line with the criteria in Table 2. Results from between 1971 and 2008 were screened by SP via title/abstract looking for high relevance papers only. Included papers were then sorted into groups (dates published; relevance category) and labelled for further analysis. A sample of 10 papers in each category was discussed with other reviewers (SS, CP, JM) until consensus was achieved.

**Table 2:** Inclusion and exclusion criteria used in selecting studies for review

Inclusion criteria	Exclusion criteria
Empirical studies that: a) use case study methodology, including as part of a wider study (e.g. RCT) b) involve any health-related intervention that may be referred to as complex at the intervention or system level in any setting (e.g. hospitals, community) c) involve any population/participants in any part of the world d) focus on health care and related topics (e.g. information systems, management, public health interventions) Other papers focused on case study and complex interventions that draw on empirical examples but are not original research papers.	Empirical studies that: a) involve animals or were not on humans b) are not published in English c) cover specific topics (e.g. tourism, farming) and study types (e.g. cost-effectiveness models, protocols or simulations) that were not relevant to our review d) do not evaluate an intervention e) do not explicitly refer to use of case study design and/or methods or present ‘illustrative case studies’ (summarising a topic via a series of cases) f) whole-country case studies (unless specific case studies of national-level institutions, e.g. departments of health, in which global health interventions were taking place) g) protocols-only publications Other papers presenting clinical case reports of single individuals.

Guided by the principles of meta-narrative review (Table 1) we classified papers as high, partial and low relevance based on the criteria in Table 3. High relevance papers explicitly focussed on all the 3Cs, i.e. case study, context and complexity.

**Table 3:** Criteria for classifying papers according to relevance

Relevance	Main criteria	Additional criteria
High	Explicitly mentions and focuses on all three topics (3Cs) of case study, context and complex health interventions	Mentions 2Cs PLUS it: shows depth and explanation of ‘case’; may have theory relevant to context and/or complexity
Partial	Mentions 3Cs but appears to give very little detail	Close reading of methods does not give clear indication of focus or depth
Low	A case study of a complex intervention (2Cs)	The description or the research means there are questions over overall quality or relevance to questions of context, complexity or case study methodology

Cycle 3 involved identifying and reviewing: (a) additional seminal, methodological texts that were cited in high relevance empirical papers; and (b) ‘hybrid papers’ focused on the merits and challenges of case study in the evaluation of complex interventions that were identified during Cycle 1 and/or were cited in high relevance empirical studies (signalling common logics) and made reference to context and complexity. Hybrid papers provided methodological touchpoints enabling researchers to connect with key issues in case study methodology. Written by scholars from a particular discipline who advocate for the use of case study for evaluating interventions in their own field, hybrid papers frequently condensed original methodological texts (e.g. Stake, Yin), using (mostly their own) empirical examples to illustrate case study research. They sometimes (but not always) included a set of quality and/or reporting criteria.

### Data extraction and analysis

Our primary focus was on empirical studies (Cycle 2), with methodological texts (Cycles 1 and 3) enabling us to question how researchers had operationalised case study approaches.

Giving more weight to high relevance papers, we analysed papers in reverse chronological order: starting with 2015–2019, working backwards to 1971 and monitoring how case study research for complex interventions was reported within traditions over time. We sampled 8–10 papers from each time period (71 empirical papers and 8 hybrid papers = 79 in total). The review team (SP, SS, CP, JM) discussed these in detail (e.g. focusing on epistemological underpinnings, how case study methodology was invoked, relevance of context and complexity). We summarised key aspects of each study in a data extraction spreadsheet (e.g. disciplines; key and additional methodologists cited; definition of context; definition of case; discussion on complexity; data collection methods; findings on context). The spreadsheet was modified as we read more papers, with the process then repeated for earlier papers. Adopting a hermeneutic approach, enabling ‘dialogue between the reader and the text, between readers and between texts […and…] translation in a concrete socio-historical and cultural context’ ([38] p262), we explored key concepts, epistemologies and methods within and across papers, both within particular traditions over time and across traditions.

Guided by a further set of analytical questions inspired by methodological texts (e.g. what does the case study do in this instance? what is this case a case of? how is context operationalised? how is context discussed in relation to the intervention? where does complexity lie according to the author?) we then deliberately placed papers in dialogue with one another. We did this by reading sets of papers each (with SP also reading all papers), sharing analytical notes and meeting regularly to discuss and refine, paying attention to the periods the sets were coming from and other connections (e.g. cross-citations) amongst them. We focussed on narrative threads (i.e. the ways in which authors tell stories about case study, context and complexity to the reader and to each other) to sensitise us to authors’ discussions that ran across groups of studies, allowing us to summarise the assumptions and values driving the empirical research. These narrative threads (e.g. about generalisability of findings; or what made an intervention complex) showed both commonalities and contradictions across research traditions. Indeed, in some cases contradictory narrative threads were evident in the same paper. For example, some studies did not fit neatly into a recognisable methodological paradigm, others recognised context in a specific way (e.g. as emergent) but then failed to operationalise it that way. This process led us to build a set of descriptive statements about the use of case study research and understandings about context and complexity that, in turn, helped us to obtain a picture of the different meta-narratives present in the literature.

We presented emerging narrative threads and meta-narratives to the wider team (JG, MP, and TG), and colleagues (e.g. seminars), with their feedback informing further analytic work (e.g. returning to methodological texts to appreciate threads).

## Results

### Summary of search results and overview of the literature

Search results are presented in Fig. 1. The total number of texts informing the review was 139 (71 empirical, 8 hybrid and 60 focusing on case study methodology). Most research teams reporting empirical studies were based in the United Kingdom, followed by the United States, conducting research in the same countries. Fewer study teams were based in Canada, Australia, and in sub-Saharan African and European countries. Authors typically worked in health services, health systems, population health, public health and primary care research teams.

Case study research spans several fields and encompasses multiple perspectives that are grounded in different assumptions about the nature of reality and lead to different combinations of methods applied in different ways [22, 39]. This epistemological and methodological diversity was reflected in the empirical case studies reviewed, which covered a wide range of case study designs, from naturalistic approaches (typically employing qualitative methods and focused on one or a small number of cases) to more quasi-experimental studies (typically employing mixed methods across a larger number of cases and with some attempt to standardise aspects of the design across cases). In almost all papers in our dataset, authors placed more emphasis on procedural aspects of the methods and tools used (e.g. data collection, sampling) than on discussions of epistemology or methodology or on the nature, selection, definition or boundaries (if any) of ‘the case’.

### Four meta-narratives reflecting four distinct research traditions

We identified four broad meta-narratives on case study research, context and complexity (Table 4). We summarise each below before examining commonalities, debates and tensions across these traditions. It should be noted that whereas two of the meta-narratives (1 and 3) were fairly distinct, meta-narratives 2 and 4 showed some overlap which reflected cross-fertilisation of ideas between them. Of note, as well as the four meta-narratives, we identified an additional set of papers that were classified as ‘case study’ (e.g. in the title or abstract), but on closer reading appeared to be qualitative or mixed-methods studies addressing context and complex interventions that were not designed to be case study research and did not engage with case study methodology. We highlight this set of papers in our Discussion as they reveal an important issue of classification and reporting of study research.

**Table 4:** Meta-narratives on case study, context and complex intervention identified in the empirical literature

	Meta-narrative #1: Case studies develop and test complex interventions [40–49]	Meta-narrative #2: Case studies analyse change in organisations [50–54]	Meta-narrative #3: Case studies are appropriate for conducting realist evaluation [55–60]	Meta-narrative #4: Case studies enable naturalistic study of complex change [61–65]
Epistemological assumptions	Positivist	Positivist or critical realist	Social realist (realist evaluation)	Interpretivist, critical realist
Case study approach	Procedural – case study as enabling testing of complex interventions in ‘real life’ contexts	Recursive - in-depth study in which theory and data are mutually reinforcing	Structured - interrogating how mechanisms triggered in specific contexts lead to particular outcomes	Iterative - naturalistic, emergent, reflexive, and using theory-building to surface complexity
Data collection methods	Main phase predominantly quantitative. Qualitative methods used to develop the intervention and provide triangulation	Purposive collection of qualitative and quantitative data to build and test theory	Semi-structured interviews to surface theories of change to guide collection and analysis of qualitative and quantitative data to explore these	Predominantly qualitative, with strong anthropological emphasis (e.g. ethnography) and high degree of researcher reflexivity
Approach to theorisation	Seeking to identify contextual factors that affect intervention outcomes, and measure their contribution	Developing and extending middle-range or programme theory about the forces that drive change in organisations.	Developing middle-range or programme theory, perhaps expressed as context-mechanism-outcome (CMO) relationships	Rich description of the case is foregrounded with use of substantive theory to inform and extend analysis
How complexity is articulated	Mechanistically, as multiple mediators and moderators of the effect of the intervention	Dynamically: the system is seen as evolving over time	Generatively, as an intervention triggers different mechanisms in different contexts	Narratively, as the non-linear unfolding of events and actions, including adaptation to change
How context is conceptualised	Characteristics of the implementation setting or human factors impacting the intervention	Dynamic organisational, policy or human backdrop, changing over time as the intervention is implemented	Set of circumstances where particular mechanisms are triggered to produce particular outcomes	Emergent and co-shaped through relationships and wider social influences on implementation practices
Analytic approach	Primarily deductive, applying frameworks to aggregate data from multiple sources	Comparative logic of analysis; sometimes informed by a priori themes from earlier phases of case study	Retroductive logic, centred around context-mechanism-outcome formations	Iterative, involving reflexivity, interpretation, multiple kinds of data synthesis and (sometimes) dialogue with theory
Basis for transferability	Theoretical replication	Primarily interest is in the case/s, may develop middle-range theory	Identification of (generative) causal relationships: demi-regularities	Naturalistic or theoretical generalisation
Outputs style	Typically, a structured academic paper, including a diagrammatic model of links between intervention and outcome	Varied, but often narratively as a long and discursive report. Some are more structured, offering a series of hypotheses that have been tested	Methodological narrative which sets out how different configurations of context and mechanism were systematically tested and confirmed or rejected	Rich case narrative which highlights the unique detail of the case and may use literary devices (e.g. metaphor, surprise) to create a compelling story

### Meta-narrative #1: Case studies develop and test complex interventions

This first research tradition presents the case study as a way of testing complex interventions, comprising a set of specific instructions on how to design, conduct and report on a case study (Table 4). Building on the Medical Research Council’s widely cited Framework for Developing and Testing Complex Interventions [9], this tradition favours a qualitative development phase followed by a comparative case study testing phase, as illustrated by the early SHIP study, which formed a model which others followed and refined [47]. Case study research in this tradition is based on broadly positivist assumptions and the ‘theoretical replication’ methodology of Robert Yin. The focus is on technical research approaches and methods designed to test hypotheses about the impact of an intervention (and what mediates or moderates it) in real-life contexts. Researchers identify a pre-existing case or series of cases (e.g. one or more hospitals) and a specific intervention (e.g. an improvement effort to reduce waiting times), then set out to identify relevant contextual factors that are pre-existing and independent of the intervention (e.g. case mix, technological innovativeness) that can explain variations observed between the stated intervention objectives and outcomes in different settings. Complexity is viewed as an inherent property of the intervention or the context in which it is implemented.

In this tradition, case study research is regarded as an appropriate research design because it offers a robust and transparent research procedure for answering questions about ‘how’ and ‘why’ the intervention works in a specific setting, community or population. Take the example of a case study of an equity-enhancement intervention in primary care in Canada – researchers deliberately used case study as ‘*a comprehensive research strategy useful in exploring, describing, explaining, and evaluating causal links in real world interventions that are too complex to be assessed by survey or experimental strategies alone*’ ([44] p7).

Case study research that focuses on testing complex interventions often claims to use mixed (qualitative and quantitative) methods. However, data collection methods are predominantly qualitative (e.g. interviews, focus groups, documentary review), and quantitative data tend to be used as an illustration (e.g. describing a reduction in waiting time as part of a wider narrative of improved efficiency) [41]. The use of multiple data sources is frequently given as evidence of a case study approach, and such “data triangulation” is greatly valued as a way to increase the reliability of case study findings. The analytic process (e.g. framework analysis) is usually deductive, based on aggregation and commonly synthesised in a ‘case description’. For instance, a study of improvement efforts to ameliorate hip-fracture care at a Swedish acute care hospital used a stepwise approach in which data were ‘*organized and coded to characterize a) process problems (before and during the redesign), b) the actual changes carried out, and c) the effects of changes as reported by staff members*’ ([41] p3). Other studies used structured analytic techniques e.g. framework (see, e.g. [40]). Guided by Yin [30], multiple case studies typically present within-case followed by cross-case analysis, seeking generalisation of findings through a process of aggregation.

In this research tradition, the term ‘context’ is not defined but usually features in the commonly cited definition of what a case study is: *‘an empirical inquiry that investigates a contemporary phenomenon within its real-life context’* ([66] p13). The focus is on defined and tangible contextual features external to the intervention. For example, in a study of the implementation of public health policy in two Swedish municipalities, researchers examined ‘*the contextual steering mechanisms that are practiced in local government*’ ([43] p220) and local government implementation of national policies – both were held as conceptually separate, with opportunities to act and implement national targets *‘restricted by surrounding structures*’ (p221).

Context is often referred to in terms of specific ‘contextual *factors*’, which are typically framed as background to the implementation of an intervention and articulated in terms of a (heterogeneous) list of pre-existing features. For example a case study of an equity-enhancement intervention in primary care listed 5 ‘*contextual factors*’ that shape the intervention: ‘(*a) the characteristics of the population; (b) the characteristics of the staff; (c) the organizational milieu, including formal and informal power structures, policies, and funding; (d) the political, policy and economic context (…); and (e) the historical and geographic context, specifically, the physical location of organizations in varied rural and urban locations, and the social conditions linked to those locations’* ([44] p7).

In this tradition, study descriptions of context are often narrower than the contextual factors considered, and provided as rationales for case selection or sampling. For example, in the above mentioned Swedish study, two different municipalities were selected as cases to illustrate different local contexts, with specific characteristics of each municipal organisation then described as ‘contextual conditions’ [43].

Complexity (e.g. of the system, setting or intervention) is sometimes invoked in studies in this tradition as a rationale for selecting a case study design (e.g. [44]) or is presented as a characteristic of the case. But whilst complexity is often mentioned by name, the narrative thread on complexity is typically thin and scarce, often appearing as fleeting statements that reveal little as to how complexity is understood. Complexity, it seems, is just *there* but does not have to be theorised. The linear nature of the case study methodology in this tradition constrains opportunities to engage with complexity. Hence, while an interest in underlying intervention mechanisms is evident in the decision to adopt case study methodology, this typically plays out with researchers then deconstructing the phenomena under study into factors, components or levels in order to describe associations between context and impact on interventions.

The knowledge produced from designs in this tradition is mostly descriptive, presented in technical accounts detailing contextual factors affecting the intervention. Where theory is drawn on, this is for the specific purpose of disentangling the mechanisms through which the intervention operates in the case study. For example, in the study of hip-fracture care, authors do not cite a particular theoretical approach. They simply state that intervention complexity and the heterogeneity of intervention ‘*application*’ in different contexts ‘*constrain generalizations about which method works, when, and how. To gain a deeper understanding of what works, research needs to better disentangle what is actually being implemented and how the multiple components of improvement interventions contribute, or do not, to improved operational performance*’ ([41] p2).

The relationship between context and intervention (where addressed) tends to be fixed, with intervention success or failure explained as a matter of ‘fit’ between the relevant theory or hypothesis behind the intervention and the context of implementation. Variation between the local contexts of cases accounts for differences in implementation processes and outcomes. For example, a study of the introduction of an electronic audit and feedback system to improve maternal-newborn care practices and outcomes found that a ‘*one size fits all approach*’ was not feasible because ‘*the diversity in context within our case hospitals and in the facilitators and barriers they experienced demonstrates the challenges of implementing one audit and feedback system across an entire sector (all maternal-newborn hospitals)*’ ([45] p641).

### Meta-narrative #2: Case studies analyse change in organisations

Theory-informed case studies of organisational and institutional change, including quality improvement efforts, seek to understand and evaluate the practices, processes and relationships relevant to the development, implementation and adoption of an intervention within specific organisations (Table 4). This tradition is more heterogeneous than the one described in meta-narrative 1, having more dispersed origins and wider influence from outside health services research. Researchers in this tradition share a commitment to bringing theoretically-informed rigour to the empirical study of organisational change and quality improvement. Whilst the case study methodologists cited vary, many studies in this tradition are inspired by the work of Yin [30] and adopt a positivist or critical realist perspective in which the ‘real world’ is external to the intervention. Evaluation involves testing relevant theory (sometimes referred to as a programme theory – that is, overarching theory of how an intervention is expected to work and its anticipated impacts). The unit of analysis is almost always the organisation (or department), and researchers use multiple data collection strategies to study, for instance, how staff variably perceive and carry out change-related activities (e.g. use a new computer system [52] or create partnerships to sustain organisational innovation [50]). Theoretical constructs such as agency (which varies amongst actors) are explored in evaluating planned changes, enabling researchers to account for power and resistance in organisations.

Within this broad tradition, there are some differences with regards to what research is valued by scientists in the tradition and how it should be done. One is a system approach to patient safety, in which medical error is theorised as emerging not primarily from individual failings but from features of the system (which is seen as complex and dynamic) [52, 54]. Another approach, in which patient safety is also a prominent theme, considers how technologies (used and creatively adapted by humans) subtly alter both front-line work practices and the behaviour of the wider system (e.g. creating panopticon-like surveillance of staff) [51]. In each of these ‘sub-traditions’, successive studies seek to test and refine theoretical explanations of organisational change generated by previous authors.

Narrative threads about case study research in this tradition, portrayed the case study as an opportunity to study in-depth organisational practices and relationships and develop theories about how these change over time. The case - and what it is a case *of* - is rarely defined and can sometimes be conflated with setting or organisation of interest (e.g. a hospital). The selection, rather than definition, of case or setting is sometimes explained. For example, in a study of automation in drug-dispensation, the authors illustrate an ‘*archetypal case (…of…) failure*’ of such an innovation carried out in an ‘*ongoing field of activity’*, i.e. a busy emergency department in a US hospital ([52] p1494).

Qualitative data is usually collected via interviews, observation and documents analysed using the constant comparative method, with authors sometimes reporting using a priori themes from pilot/exploratory phase or literature review. The analysis reveals differentiated intervention effects through the interpretations of different actors involved and inherent consensus and tension.

Context is not defined, but is operationalised through detailed description of the organisation and macro-level changes that frame the intervention. For example, in a study of transformational change of multiple healthcare services into a single regional service in Australia, the context for evaluating ‘*the micro detail of healthcare reform processes*’ was made up of the ‘*forces that influence* [the] *nature of change efforts in the healthcare sector*’ ([53] p33).

The focus on specific organisations has led in this tradition to a heterogeneous approach to context. Researchers frequently equate case study setting (e.g. hospital) not only with ‘case’ but also with context, and/or focus on local and national policy contexts (commonly funding issues) as ‘external’ conditions shaping ‘internal’ change efforts. For example, returning to the above-cited study, authors continue: ‘*the change in policy direction (...) was an event that occurred outside the control of the project and an example of the way in which public sector agencies are subject to change caused by the political context within which they operate’* ([43] p39).

In addition to description of external conditions, context was also operationalised through detailed description of the characteristics of actors (e.g. level of buy-in; power differences), organisations (e.g. management structure, organisational culture), and relationships amongst staff in organisations, as well as of the intervention and its origins (e.g. a study of the use of secondary data analysis from an electronic patient record system to improve safety and quality of care in a UK hospital offered information on a decade-long timeline of the introduction of the e-database itself [51]). Case study thereby enabled in-depth analysis of one or more units and description of ‘internal’ contextual differences, tensions and contradictions. In contrast with meta-narrative 1, some aspects of context that were internal to the case study organisation were shown as dynamic. This was due to relationships between staff or stakeholders being altered by the intervention.

In this research tradition, there is a noticeable interest in complexity, particularly relating to issues of diffusion, adaptation, implementation stages or cycles of the intervention, as well as sustainability, change and contextual shifts over time. Complexity is captured in iterative methodological approaches to case study evaluation. In a paper reflecting on a case study of healthcare reform in Australia, the authors adopt multiple methods across multiple levels of the system, citing the need *‘to ensure that the evaluation has the flexibility and breadth to accommodate a changing and complex context’* ([67] p492). The form of data, the sample and the overall structure of the research designs within specific organisations still tends to be pre-determined a priori, but there is room for adaptation over time.

The knowledge produced draws out positive and negative effects of interventions, often through the lens of the different actors involved. Intervention and context remain separate. The intervention is framed as a set of prespecified activities and processes that are re-interpreted by staff at different levels in the organisation.

Accounts provide the detail of intervention effects through actors’ eyes and intrinsic aspects of change in study sites. Empirical generalisation is not a primary objective, hence there is often limited exploration of how findings might be relevant to other settings. For example, in one study of safety improvement programs in US hospitals, the authors set out how their methods were intended *‘to capture a snapshot of the key accomplishments of leading organizations and to synthesize the self-perceived learning of their internal change leaders*’, rather than being ‘*meant to be representative of all health care organizations*’ ([54] p166). However, theoretical generalisation through development of middle-range theory is often an explicit objective, allowing transferability of theoretical findings (see, e.g. [51]).

### Meta-narrative #3: Case studies are appropriate for undertaking realist evaluation

Case studies in this research tradition apply the theories and methods of realist evaluation [68], which interrogates how intervention outcomes are achieved by mechanisms triggered in specific contexts by systematically formulating CMO (context-mechanism-outcome) configurations (Table 4). The realist evaluation tradition drew explicitly on social realist philosophy and the foundational work of Pawson and Tilley who originally developed the approach within social policy research [68]. A seminal paper in 2005 made this work accessible and appealing to health services researchers [69]. A leading research funder, the UK National Institute of Health Research, was attracted to its systematic approach to exploring why interventions work well in some contexts but less well in others, and supported the development of guidance and standards (‘RAMESES’) for both empirical studies and theory-driven systematic reviews of such studies [36, 70]. Many though not all studies in our sample followed the RAMESES methods and reporting structure and were ‘realist’ in the sense meant by Pawson and Tilley: surfacing policymakers’ theories about why a programme was thought to work, then testing these theories by collecting and analysing (mostly qualitative) data. The main empirical phase maps context-mechanism-outcome configurations as emerging from data analysis, and identifies (generative) causal relationships in order to develop middle-range or programme theory that can account for how and why an intervention works (or not) and under what conditions. Some studies that cited Pawson’s work appeared to be realist in name only or to be based on a different conception of realism known as critical realism (developed and popularised by Bhaskar). Within this meta-narrative, therefore, not all studies followed the methods that have been endorsed by scholars in the tradition.

Realist case study evaluation is seen as a theory-testing approach because the case study can ‘*illuminate mechanism in relation to outcome*’ ([60] p5). Case study methodology is advocated due to the focus on phenomena (e.g. interventions) in context, linking closely with the emphasis in realist evaluations on ‘*how causal mechanisms are shaped and constrained by social, political, economic (and so on) contexts*’ ([70] p9). The choice to use case study is often because it allows multiple and emergent data collection methods (e.g. [58, 60]). For example, one paper reporting trial of a breastfeeding support group in Scotland describes how realist evaluation ‘*examines baseline contexts, how organisations, structures and interrelationships shape both implementation and outcomes over time’* ([58] p771). Authors reflect that it is ‘*detailed case studies, employing quantitative and qualitative data’,* that are useful in order *‘to test our propositions about the importance of context, organisation and professional relationships for outcome’* (p. 771)*.*

Case studies in the realist evaluation tradition commonly employed qualitative data collection methods (e.g. interviews, focus groups, observation) supplemented by targeted quantitative methods (e.g. structured surveys, retrospective cohort data). Different data were generally analysed separately first, using deductive and inductive approaches. Findings were then synthesised to map context-mechanism-outcome formations, using retroductive logic (i.e. asking “what could explain this?” and building and testing hypotheses about mechanisms that produce what are known as ‘demi-regularities—things that *tend to* happen, though they do not *always* happen, under particular circumstances). Counterfactual thinking (“what if this were not the case?” or “what if this happened instead of that?”) is used to test alternative explanations that can confirm or disprove the context-mechanism-outcome hypotheses obtained.

The notion of context features strongly in the realist evaluation tradition. It is central to the theoretical core of realism and viewed as a set of circumstances where mechanisms are triggered to produce specific outcomes. However, as noted above, broad definitions of context included in theoretical and methodological papers did not always match how context was understood or operationalised in the studies reported. The meaning of context was wide-ranging, capturing the characteristics of organisations or local area, relationships between staff or broader regional or national policies. Context was also linked to ‘space and place’ with, for instance ‘*the public–private interface and tensions between a mother’s choice and societal pressures’* ([58] p769) used as a starting point for theoretical development.

Some authors discussed the challenge of differentiating context from phenomena when seeking to distinguish mechanisms. This is illustrated in a case study of a mental health intervention to improve links between primary care and specialist services in England [55]. Here prevalence of mental health conditions, GPs’ professional background, or the relationships between staff could all count as context, with authors reflecting that: ‘*the same phenomenon could be coded as an outcome or context, or as an outcome or a mechanism. For example a disease register was an outcome of service development and could then act as a mechanism for improving care*’ (p.78).

Realist case study evaluations tended to include two key narrative threads about complexity, both shaped by an understanding of the interaction between context and mechanisms in the production of intervention outcomes. First, that realist evaluation is an appropriate approach to understanding complex interventions (i.e., the intervention is complex in and of itself and realist evaluation can unpack its differential outcomes). Second, that the complexity of the intervention is surfaced in the implementation context or system (i.e. complexity can be observed through the evaluation as it emerges from the interaction between intervention and context). For example, in a case study of academic/practice collaborations in England, authors suggest that: ‘*Realist evaluation is particularly appropriate for developing explanations about how programmes, which by their nature are complex, work contingently within the context of implementation*’ ([59] p3). The realist case study approach is presented by authors as ideal for exploring this complexity, allowing researchers to study ‘*[collaborations] that are complex, in the sense that their behaviour can be explained with reference to the properties of a whole (adaptive) system rather than its individual components’* (p. 13). The authors conclude that such an approach *‘enables a complexity theory lens that views outcomes as emerging from interactions amongst individuals within a system’* (p.13).

In terms of the knowledge produced, outputs of realist evaluations tend to be presented as technical reports focused on how and why the intervention did (or perhaps didn’t) work. In-depth, thick description and rich information on context are required in order to obtain ‘*insights into the attributes necessary within complex health systems for a policy to work*’ ([58] p777). As Byng et al. [55] report: ‘*in some cases potential contingent mechanisms or contexts could not be identified to explain why a mechanism was associated with an outcome in some situations but not others. This could be due to the paucity of data regarding potential contingent contexts or due to inconsistency of the data and lack of clear associations’* (p.79). There is a sense, however, that in such cases the researchers felt that they had looked exhaustively for CMO configurations and identified all the demi-regularities there were to find.

### Meta-narrative #4: Case studies enable naturalistic study of complex change

This research tradition, inspired by the work of Robert Stake [71, 72], is oriented to achieving hermeneutic understanding and is characterised by a deliberately open-ended approach to the case, complexity and context (Table 4). Grounded in interpretivism (an orientation to inquiry that sees social reality as shaped by human experiences and social contexts), this kind of case study research involves granular, naturalistic and often longitudinal observation of events and relationships. The detail of the case is built iteratively, with context understood as an emergent property of ongoing interactions between the complex system and intervention. The researchers’ task is to interpret these interactions though a process of sense-making. Some researchers in this tradition (but not all) seek to engage with, and extend, social science theory.

Whilst ‘thick description’ (that is, a very detailed presentation of real-world events and settings using the narrative form, illustrated with extensive extracts from field notes and on-the-job interviews) is valued to some extent by all case study researchers, in this meta-narrative such description is a goal in its own right. In this sense the naturalistic case study can trace its origins to seminal work in anthropology, where thick description was advocated to provide a picture of what human behaviour and symbols meant in different cultures so they could be *understood* [73].

Unlike the traditions described in meta-narratives 1–3, the design of naturalistic case study research is not prescribed. The effects of interventions are seen as nonlinear, explained by narrative causality, as in the events in an unfolding story. Instead of seeking predictable and generalisable relationships between variables (as in meta-narrative 1), transferable theoretical models about change (as in meta-narrative 2) or demi-regularities (as in meta-narrative 3), this tradition is oriented to describing ‘*interacting processes [and the] extent of reciprocal adaptation and embedding*’ ([61] p539). The focus is on an emic (i.e. from the participants’ perspective) analysis of the case (as opposed to an external, “etic” analysis from the researchers’ perspective), with selection of data sources guided by the principle of ‘the opportunity to learn’ [74]. Researchers are interested in reflexivity, granularity and preserving ‘multiple realities’—that is, the perspectival view of different individuals and interest groups, which may conflict but which, taken together, contribute to a rich picture of what is going on ([72] p12). In sum, naturalistic case study research is understood as building a rich, detailed picture in context. In this tradition, the understanding of a case is not specified up front and emerges through the process of conducting the case study.

Data tend to be gathered via qualitative, especially ethnographic, methods (e.g. observation), sometimes with additional quantitative data such as clinic audits relating to patient outcomes and demographics, and patient surveys (e.g. [58]). Longitudinal approaches are favoured. Another important data source is the reflexive experiences of the researcher, which may include accounts of events ‘from the field’ and an analysis of the researcher’s reactions to these (what John Van Maanen calls ‘confessional tales’ [75]). In contrast with the research tradition described in meta-narrative 1, where the researcher is seen, more or less, as a dispassionate observer in the case, in this tradition he or she may, in some cases, be a character in the story of the case study. Analysis of the dataset in naturalistic case study involves iteration, comparison and integration of multiple data sources using narrative as the key synthesising device, an emphasis on stakeholders’ perspectives, input from those involved in the research and (in most cases) an ongoing dialogue with relevant theory. Reflexivity is seen by some authors as aiding transparency about subject position and relational dynamics between researcher and researched.

Context is rarely defined in this research tradition, but represented as emerging from a set of relationships and in interaction with wider social forces (e.g. the economy). Such interaction is situated as ‘sense-making’. To put it another way, the essential goal of naturalistic case study is to tell a story, and the story form presents actions and their contexts as interwoven. Operationalisation of context emerges through a narrative iteration between micro and macro contexts and through reflexivity (which brings in the context *of the researcher* as well as the research). Whilst naturalistic case study has traditionally placed little emphasis on theory (emphasising what Stake has called “the intrinsic study of the valued particular” ([76], p448), those who have applied this approach in a healthcare setting have often brought in theoretical models to move back and forth from the particular of the case to the general lessons that might be drawn from it (e.g. [62, 65]).

Naturalistic cases are usually singular, with the knowledge produced revealing complexity through thick description of complex processes and systems. Meta-level accounts of problems and solutions read as accounts of the particular, with close analysis of the specificities of each case instrumental in generating in-depth understandings about wider structural relations and the unfolding of complex change that are potentially illuminating for (though not predictive of) other situations and settings. Cases do not need to be representative to learn from and generate knowledge [73, 77]. The basis for transferability is primarily *naturalistic generalisation* (in which the researcher and those who immerse themselves in the detail of the case acquire a richer vocabulary and imaginative capabilities which they can then apply to other cases), and—to a lesser extent—*theoretical generalisation*, where the rich description of the case enables the application of theory, potentially increasing the explanatory power of the case [76]. Most of the naturalistic case studies in our dataset favoured rich description without extensive theorising. For example a community HIV project in South Africa [63] was presented without use of the word ‘theory’; a study of hospital mergers in the UK [62] states that a ‘preliminary theoretical framework’ was selected to guide data collection but is not mentioned further in the paper. In a study of the sustainability of whole-system change in healthcare in London, a theoretical framework based on system dynamics ‘*was developed after completion of the data collection’* and used to inform analysis ([61] p542). In all three cases, however, the primary focus of the paper is on presenting an authentic descriptive account.

### Commonalities, debates and tensions across meta-narratives

#### Engagement with methodological literature

Two key methodologists – Yin and Stake (Table 5) – were repeatedly cited across empirical papers. Those adopting a ‘Stakian’ approach differed, often significantly, from those drawing mainly on Yin, though it should be noted that many studies cited these methodologists without following the actual methods they advocated (some studies in meta-narrative 1, for example, cited Stake but approached case study research from a technical and largely positivist stance). Both Yin and Stake (and also Pawson, who draws broadly on Yin) emphasise detail, depth and contextualisation; however, while Stake’s method aims to build a naturalistic and evolving picture in context through immersion and interpretation, Yin’s pays more attention to design choices at the outset (e.g. case selection, sampling), a priori theoretical frameworks, and the description of step-wise processes (e.g. to develop chains of evidence). This distinction was reflected in our review. Yin-influenced studies (meta-narratives 1 and 3 along with most studies in 2) tended to describe and justify certain elements of case study design (e.g. the type of case study; data collection methods) more than others (e.g. definition of the case). Studies inspired by Stake (meta-narrative 4) tended to emphasise knowledge as emergent.

**Table 5:** Overview of Robert Yin’s and Robert Stake’s work on case study research

Yin’s *Case study research: design and methods* was first published in 1984 [30]. It provides a step-by-step guide to conducting a case study and has been cited in scholarly literature over 44,000 times. His largely positivist approach emphasises a priori design and theoretical frameworks, and a drive to examine causality through analytic generalisation and naturalistic inquiry. Linked to a renewed interest in case study in health services research from the 1990’s onwards, Yin’s work has been taken up by different research traditions in health care (e.g. nursing). His approach has attracted interest for the evaluation of health interventions in instances where experimental design have been seen as unfeasible or unethical.	
Stake’s 1995 book on *The Art of Case Study Research* [72] focuses on qualitative case study methodology underpinned by a constructivist standpoint in which ‘*knowledge is constructed rather than discovered*’ (p.99). Stake’s focus is on the particular, understood in context with case study enabling researchers to study, in detail, the particularity and complexity of a single case and ‘*coming to understand its activity within important circumstances’* (p.xi) According to Stake, multiple interpretations, including those of the researcher/s, are involved in the construction of knowledge about the case.	

As we synthesized findings across the literature in Cycles 1–3, we were struck by many authors’ limited reflexivity as to how case study methodology was taken up and modified in empirical application, leading to multiple, contradictory and confusing narrative threads about the philosophical foundations and methodological requirements of case study research. For example, empirical case studies in meta-narratives 1 and 2 tended to approach case study research as a set of procedures or tools. Few of the studies citing Yin (especially those in meta-narrative 1) included an explicit theoretical and methodological aim, despite Yin’s emphasis on setting out theoretical propositions a priori. There was an inconsistent use of Yin’s original methodology.

Some studies used ‘hybrid papers’ (Table 6) as a source of methodological input. Table 6 shows key observations of relevance in these papers with regards to the study of context in complex interventions, and the meta-narratives they relate to.

**Table 6:** Overview of hybrid papers (Cycle 3)

Examples	Aim of paper	Citations^**a**^	Key influences	Disciplinary home	Key messages for 3C	Key MNs^**b**^
Bergen A et al. A case for case studies [78]	To explore relevance of CS concepts to nursing research	220	Yin	Nursing	It is necessary to analyse case and context data; internal and external validity strengthened e.g. via theory building	1
Crowe et al. The case study approach [79]	To introduce and assess different CS approaches, questions and methods	1627	Stake and Yin	Primary Care/ Population Health	Suggestions to address concerns about transferability, incl. Theoretical sampling respondent validation and transparency	1,2
Payne et al. Case study research methods in end-of-life care [80]	To advocate for CS research in nursing and to summarise practical issues	97	Yin (Stake mentioned)	Nursing/ Palliative Care	Case selection is central to theoretical generalisation; mentions reflexivity in knowledge production	2
Segar et al. Thinking about case studies in 3-D [81]	To reflect on methodology of their case studies of healthcare commissioning	5	Yin, Flyvbjerg	Health Services Research	Important to define boundaries of the case and to focus in-depth on place and local history to understand change	2
Sharp. The case for case studies in nursing research [82]	To set out when CS useful for studying practice, including where there is a complex pathway of care	121	Yin, Stake and Walshe	Nursing	CS have limited capacity for representativeness and generalisation (only to similar situations)	1,2
Van Eyk et al. Evaluating healthcare reform [67]	To reflect on methodological challenges of case study evaluation of healthcare reform	17	Yin	Public Health	CS should consider historical and political context to reform; research needs to be participatory and flexible to adapt to change during evaluation	2
Walshe C. The evaluation of complex interventions in palliative care [25]	To advocate for CS to address complexity of interventions	74	Stake, Merriam, Yin, Flyvbjerg, Walshe	Palliative Care	More emphasis needed on case selection, longitudinal designs, and use of rival hypotheses to enhance study of complexity	1,2
Wells et al. Intervention description is not enough [28]	To examine the relationship between context and intervention	207	Yin	Nursing	Context and intervention are co-constructed; complex interventions evolve during trials; such influences compromise RCTs	1

^a^ from Google Scholar; ^b^ 1 = Case studies develop and test complex interventions; 2 = Case studies analyse change in organisations

This body of hybrid literature was important in providing a ‘bridge’ from the empirical work to methodological sources. However, they frequently provided selected methodological detail (likely due to space constraints as journal articles) and tended to draw predominantly on Yin and Stake. There was limited reference, in empirical and hybrid papers, to how other disciplines have engaged with context and with the case, and how this has informed the researchers’ understanding of complexity in their study. This carries the potential to narrow the scope and potential of the methodology.

Overall, leading case study methodology experts from outside the healthcare field (e.g. Mitchell [83], Gerring [84, 85], Flyvberg [22], Burawoy [85]) were conspicuously absent in the review of empirical case studies. Even where Yin and Stake were cited, empirical papers were not always faithful to the methodological principles of the original or provided a rationale for divergence.

#### Defining the case

Case definition is central to case study research and consequential for the knowledge produced. Moreover, case selection and its intended relationship to a broader class of phenomena forms the basis for causal inference. According to Gerring, ‘*what differentiates the case study from cross-unit study is its way of defining cases, not its analysis of those cases or its method of modelling causal relations’* ([84] p353).

Many papers offered a description of how a case was selected but not of how the case under study (regardless of whether it was ‘arrived at’ a priori or with an open-ended approach) was defined. This is important as, for example, defining a case by mentioning ‘the organisation’ (as several papers in our dataset did) at the exclusion of – for instance - how policy, discourse and wider structural relations shape organisational practices inevitably limits the choice of methods, analytical approach and findings to the boundaries of that organisation. Take the example of a study of mergers between different healthcare institutions in England, based on ‘*four in-depth case studies’* [62]. Each case study focused on integration of two institutions and they purposively selected four community trusts, in which such integration was taking place (to ensure *‘range of trust types and geographical spread in London*’). Case *selection* thus appears difficult to distinguish from the sampling of units of analysis. The authors then discuss how a ‘cross-case comparison’ produces a set of themes for the paper. Their detailed account is rich and offers a sense of the different processes of integration. However it remained unclear whether there was a specific case (of the process of integration) or whether the four ‘case studies’ were rather examples of what might happen during mergers. We reflected that examples such as these raised questions about the extent to which research teams had to make discipline-related choices regarding giving much detail about case selection whilst presenting the cases themselves as having unproblematic boundaries. It may be that in the empirical reality, as the research unfolded, what was ‘in frame’ and what was ‘out of frame’ changed, but these key decisions did not make it into the paper.

#### Connecting with context

Across papers it was for the most part unclear how authors understood, approached or defined context as a concept. There were varied meanings and uses of the term in empirical case studies, with implications for how evaluations of complex interventions are designed and conducted, the knowledge produced and potential transferability.

That said, case studies in all four research traditions clearly included an intention to contextualise. This was evident in: (i) the choice of a case study approach (e.g. citing Yin’s definition of a case as a phenomenon in ‘real life context’, e.g. [40]), (ii) use of context-mechanism-outcomes frameworks, (iii) details provided about organisations or settings ‘for’ an intervention, and iv) discussion about the importance of context more broadly. Papers attempted to operationalise context in different ways, e.g. describing study settings, offering contextualised justifications for case selection, reviewing national and local policies linked to the intervention, or recounting the history of an intervention or improvement activity.

In meta-narratives 1 and 2 (where case study is often procedure-driven and context external to the intervention), papers typically engaged with context in the ‘findings’ sections by offering lists of ‘contextual factors’ to be taken into consideration when assessing the intervention (e.g. [45]). In meta-narrative 3, realist case study evaluations included ‘context’ in the construction of context-mechanism-outcome hypotheses. In meta-narrative 4, naturalistic case studies situated context as emergent, relational and in dialogue with the intervention, offering rich or ‘thick’ descriptions for the reader to gain a ‘vicarious experience’ ([72] p86) of the case and relevant context (e.g. [63]).

Strikingly, even where authors discussed the importance of context and pointed at the contexts of relevance to their study, what is meant by ‘context’ and how this applied in the empirical studies reviewed remained unspecified. This lack of clarity made it difficult to appreciate how different kinds of contexts were conceptualised, how they compare (e.g. the ‘context’ of a specific hospital versus the policy ‘context’) or the relationship between context and intervention. A handful of papers (e.g. [63] explicitly engaged with context in ways that were ontologically coherent with the methods they adopted and had a clear level of analysis to focus on (e.g. language, social action). In absence of a conceptual definition, this was helpful to make sense of contextual dimensions of the case.

Some empirical papers cited (e.g. [50, 59]) or made use of (e.g. [86]) the Consolidated Framework for Implementation Research (CFIR) [87], a meta-theoretical framework combining previous implementation research theories and models, to aid the assessment of different ‘dimensions of context’ (e.g. outer setting; inner setting) and linked sub-dimensional constructs (e.g. cost; implementation climate; planning). The papers using the CFIR largely (though not exclusively) aligned with meta-narrative 1, as the framework’s emphasis on contextual factors as ‘*surrounding the implementation efforts*’ ([87], p4) maintains a clear division between intervention, implementation and context.

Finally, a common characteristic across papers in meta-narratives 1–3 was a view of ‘changing contexts’ as an unexpected source of complexity, rather than change and dynamism being inherent qualities of context. Researchers frequently invoked the use of case study as a way to address complexity in and of context, but then revealed change as a finding. In some cases, attempts to integrate in results sections through the use of abstract phrases about ‘dynamic relationships’ were supported by limited empirical evidence of how this happens.

#### Transferability of findings from case study research

The question of transferability is central to much health services and public health research, whose goal might be said to be generating lessons from one setting that can be applied in other settings. Whilst case study research outside the healthcare field includes much discussion of this topic, we found limited engagement with the question of transferability (what some researchers call ‘external validity’) of case study evidence.

In the empirical studies we analysed, researchers rarely stated how findings could be generalised theoretically or applied to other settings. In meta-narrative 1, narratives focused on the need to aggregate and standardise datasets resulting from multiple data collection activities and provide lists of ‘contextual factors’ (typically high level and with limited contextual nuance) to explain variation in intervention outcomes. In meta-narrative 2, the focus was on how being ‘*rooted in specific context*’ means that generalisability of findings to other contexts was *‘limited by the extent to which contexts are similar*’ ([88] p9). Meta-narrative 3 used the concept of *demi-regularities* to convey the idea of partial transferability, and meta-narrative 4, as noted above, construed transferability mostly in terms of understanding and capacity to imagine, produced by immersion in the narrative detail of a single case. Overall, case studies provided insights into the organisation or other unit of analysis under study, while the choice of data collection methods, analytical approach and form of reporting meant they could easily be represented as too context specific to have wider relevance.

In meta-narratives 1–3, study findings were sometimes seen as informing middle-range theories—that is, theories that are sufficiently detailed to help explain some regularities in empirical findings but which do not account for every eventuality. For instance, studies in meta-narrative 3 sought to explain how an intervention works, why, for whom and under what conditions. This gave researchers a structured theoretical framework of reference and a way to express generalisability through middle-range theory development.

In meta-narratives 1 and 2 especially, authors frequently placed limitations on the explanatory power of single case studies (presented as offering useful points of transferability rather than stronger claims to theoretical generalisability) and appeared somewhat defensive vis-à-vis potential critiques rooted in statistical generalisation. In contrast, in meta-narrative 4 generalisation and transferability were based partly in the confidence researchers had in the naturalistic generalisability of a richly-described ‘n of 1’ case and partly on the development and refinement of substantive theory, noticing patterns, differences, commonalities or exceptions in instances or events in context and keeping these in dialogue with the theoretical approach adopted coming into the research study.

## Discussion

To our knowledge, this review is the first to focus on empirical and methodological literature relating to the intersection between case study research, context and complex interventions. Findings demonstrate the array of applications and the potential of case study research for evaluations of complex interventions in health care and public health research in key areas, including the use and refinement of theory, design flexibility and adaptability to emerging issues, breadth and depth of data sources and use of multiple methods, appreciating different kinds of causal mechanisms and complex causation, pragmatic advantages when experimenting is not feasible, and potential for transferability and generalisability from single and multiple case studies.

### Summary of key findings and links to wider literature

The review has identified four broad research traditions in which case study was used to study complex interventions and the role of context: developing and testing complex interventions; developing theoretical models of change in organisations; undertaking realist evaluations; and producing thick descriptions of the change process. In these different traditions, case study, context and complex intervention, along with the interaction between them, were operationalised differently.

In the wider methodological literature, case study research is widely recognised as an overall approach or strategy encompassing a range of methods, and places much emphasis on the understanding and definition of the case, especially whether it has boundaries and the question of what it is a case *of* [22, 71, 84, 85, 89, 90]. This was rarely reflected in the empirical literature we reviewed, with many papers emphasising case study methods but taking the case itself as given. The selection of the case (e.g. a particular intervention, a theory behind an intervention) and of the relevant units of analysis (e.g. an organisation through which the intervention is implemented) needs to be integrated with how a case study is understood and intended [81]. The lack of detail across meta-narratives about case definition and case selection (rather than simply a sampling strategy) makes it difficult to assess the value of case study evaluations. This, in turn, raised challenges for appreciating the context in which the case is situated and the evolving relationship between case, intervention and context.

Findings show that case study research offers useful avenues for analysing the nature of the relationship between context and intervention (e.g. whether distinct, interdependent, or indistinguishable). However, most empirical papers were limited in the extent to which the relationship between context and intervention was expressed and explored, the ways in which context can be understood and evaluated and the potential of case study research to aid this.

The current definition of context in MRC guidance as ‘anything external to the intervention which impedes or strengthens its effects’ ([10] p10) has been broadened to ‘any feature of the circumstances in which an intervention is implemented that may interact with the intervention to produce variation in outcomes’ in more recent Canadian Institute of Health Research/ National Institute of Health Research guidance for population health interventions studies ([13] p6). Taken together, these definitions reflect a predominant approach to thinking about context (e.g. meta-narratives 1–3), focusing on specific features that can aid understanding of any changes brought about by interaction with an intervention. Such research rarely sets out to study context, but is faced with challenges in intervention variation and understanding outcomes that lead researchers to then develop taxonomies and lists of contextual factors. This approach may risk de-contextualising context as high level ‘factors’, potentially losing the contextual nuance offered to the reader that is one of the strengths of case study research [6].

The dominance of this approach is tied up with the historical roots of health research (e.g. the biomedical institutionalisation of research and the RCT, need to generate probabilistic evidence of causality and generalisation), which has led to context being a distinct object of inquiry. By attempting to compartmentalise context/s, studies (e.g. meta-narrative 1) often move away from the stated aim of addressing complexity through case study, and return to a notion of discrete components (of the system, of the context) of a fixed reality, rather than engaging with the relational and processual nature of context and intervention.

Alongside the previously mentioned Consolidated Framework for Implementation Research [87], a number of other models of context, detailing different domains, constructs and attributes of context have become available in recent years [91]. For example, the 2017 Context and Implementation of Complex Interventions (CICI) framework is aimed at ‘simplifying and structuring complexity’ ([92] p1) in order to provide a lens for understanding interacting dimensions of context, implementation and setting. It presents a multi-level understanding of both context and implementation and sees context as ‘an overarching concept, comprising not only a physical location but also roles, interactions and relationships at multiple levels’ ([92] p6). Although not taken up by empirical papers reviewed in this paper, our scoping review showed that it is becoming popular in the field more recently.

Broader ways of thinking about and operationalising context are emerging in the healthcare field [13, 17, 27, 93] emphasising, for instance, interventions in complex social systems and even interventions as system changes in themselves [94, 95]. Methodological literature in the wider social sciences goes further, describing an array of ways in which context can be conceptualised with implications for the design, conduct and impact of case study research. There are connections across diverse disciplines between the idea of context as an event, as relational and/or socially structured action, or context as a process. For instance, Hawe’s idea of interventions as ‘events in systems’ [11], links with Rhodes and Lancaster’s idea of entanglements [96], and Meir and Dopson’s relational view as social action/process [97]. From this perspective, context is something that happens dynamically or *is performed* (usually to make sense of what is taking place) – this view of context was visible in meta-narratives 3 and 4, though not always articulated or operationalised explicitly in this way. We plan to examine cross-disciplinary contributions to understanding context in further detail in a future paper.

There are clearly opportunities for case study research to offer explanation and transfer of findings to other contexts. Empirical papers often held back from stating potential transferability and generalisability. This may be due to a historical tendency to understate and critique the potential (i) of case study to offer explanation and to test or build theory (compounded by the historical relegation of case study at the bottom of a methodological hierarchy of effectiveness) [6], and (ii) for abstraction from ‘the particular’ (e.g. specific case or context) to the general [22]. In-depth case studies, particularly in meta-narratives 3 and 4, tended to include lack of ‘representativeness’ as one of the limitations. This might be due to peer review in publication and a misunderstanding of the nature of inferences and how they are made (e.g. with authors starting with strong claims about theoretical generalisation from a single case, reviewers requesting acknowledgement of the case as limited in generalising to other cases characterised by different contextual factors, and authors accepting this point and downplaying explanatory power in order to get the paper accepted). Further work is needed to appreciate how and why transferability and inference are downplayed, how this manifests across meta-narratives and case study approaches, and to correct this.

### Strengths and limitations of this study

Reviewing a vast and disciplinarily diverse literature focused on methodology and its operationalisation was challenging. The use of meta-narrative review was critical, enabling *breadth* to identify meta-narratives and *depth* to unpick pertinent methodological, ontological and epistemological concerns. Using three hermeneutic cycles allowed us to engage with the different layers of the literature – complexity, context and case study research – in iterative ways that a standard systematic review would not have enabled.

The aim of a meta-narrative review is to connect with seminal papers and key threads running through the literature. This allowed us to foreground different disciplines and paradigmatic approaches to case study and the ways in which these shape conceptual and empirical use. As the four meta-narratives demonstrate, case study research is a broad and contested terrain, with a lack of consensus on design and implementation and variation across disciplines. Given this breadth, we were aware that there may be sections of the literature that may have used case study methodology but different terminology (i.e. would likely meet our definitions for relevance but not use the 3C terms, perhaps ones describing their work as ‘ethnographies’, ‘mixed-methods’ or ‘qualitative studies’). These articles were unlikely to have been identified through our literature search; identifying them would need a much-expanded review.

One limitation was that our search strategy was neither sensitive nor specific. On the one hand, the final dataset was relatively small, making it difficult, for example, to demonstrate historicity in the different meta-narratives. It is likely that many relevant studies were missed. On the other hand, in an attempt to avoid an over-specific search (allowing ‘2Cs’ rather than insisting on ‘3Cs’), we turned up a significant body of work that used the term ‘case study’ but did not appear to adopt the methodology and were perhaps of marginal relevance to our research question. Nevertheless, these studies comprised a significant part of the dataset and allowed us to explore the reasoning behind the reporting of evaluative research as ‘case study’.

Our review focussed on ‘intervention-dependent context’, guiding us to literature in which the primary drive to study context came from the need to understand the effects of interventions. As set out above, other ways of conceiving context are possible.

This review also focused on reports of single case studies. A key contribution of case study designs to the literature on causal inference in complex systems is through comparative analysis of series of cases, such as through formal Qualitative Comparative Analysis (QCA) methods [98]. These use set-theory to systematically compare ‘configurations of conditions’ (such as elements of context, intervention features) to identify patterns associated with presence and absence of an outcome. As the findings of a QCA study are unlikely to be reported as ‘case studies’, they would not have been in scope for our search: the ‘cases’ here are the data for analysis, rather than describing the design of the study. QCA approaches can be applied to primary or secondary data. A recent review of these methods in evaluative health research [99] identified lack of empirical diversity as a challenge: in short, better reporting of primary case studies would also bring benefits for researchers using QCA and similar methods to improve causal inferences from reviews of those studies.

## Conclusion

The need for better methods for evaluation of complex interventions is now widely recognised [13, 21, 28, 29]. The ‘complexity turn’ has drawn attention to the limitations of relying on causal inference alone for understanding whether, and under which conditions, interventions in complex systems improve health services or the public health, and what mechanisms might link interventions and outcomes [4]. The four meta-narratives identified in our review are rooted in different ways of seeing the world, leading to different case study designs and methods and the production of different types of knowledge. Clearly, there are choices to be made about the exact approach to be taken in light of the focus of any evaluation, and the research questions that users of evaluation evidence need answered, the nature of the complex intervention and the extent of complexity within the intervention and system. If evaluative health research is to move beyond the current impasse on methods for understanding interventions as interruptions in complex systems then there is a need to more fully exploit the potential learning from this breadth of case study research in evaluation of complex interventions. To do so researchers, funders and users need to address five challenges.

First health research draws on multiple, and arguably incommensurable, conceptualisations of case study research: what a case study is, and the diversity in how empirical case studies are conducted and reported. Whilst we do not believe that consistency is needed across different approaches (indeed, each has important strengths), work is needed to appreciate the range of relevant meta-narratives shaping case study research, the scope and use of different methodologies and type of knowledge produced.

Second, misconceptions remain that case study research can only provide exploratory or descriptive evidence. Yet evidence from one case can be all that is needed for causal claims (e.g. claims that X CAN lead to Y; claims that X doesn’t necessarily lead to Y). This point relates to the value given to case study research generally. However, it has a particular salience in health sciences, where the evidence based medicine movement has instilled a hierarchy of evidence in which case study is firmly relegated to the bottom of the hierarchy. While there has been challenge to this hierarchy, and some movement, resituating and internalising case study research within health sciences requires significant change in the way in which researchers, funders and publishers within the field, not only conceive and rank different kinds of evidence and different kinds of causal inference [5] and their integration (where possible), but also the ways in which the community puts that into practice (e.g. via peer review, in scaling interventions).

Third, case study researchers, especially in some traditions, typically focus on ‘thick description’ of findings as a means of contextualising detail. This can make it challenging for those more familiar with RCT and quasi-experimental approaches to evaluation to identify the key messages related to intervention evaluation. It likely requires both a readiness on the part of users (e.g. policymakers, journal editors) to engage with the detail of case study research and better skills on the part of case study authors in distilling what are likely to be key issues for decision makers.

Fourth, the relationship between *context* and *intervention* needs to be conceptualised along a spectrum, from being separate through to being in interaction. This is not simply a matter of definition, but also relates to evaluation perspective and intended utility. For instance, from a realist perspective (meta-narrative 3) one might not differentiate between context and intervention (and indeed, outcome and mechanisms), but from an intervention-centred perspective (e.g. meta-narrative 1) it might be different. This approach represents a significant challenge to current approaches that tend to construct context as a set of factors/characteristics.

Finally, papers are variably framed as case study research. This is fostered by institutionalised conventions and checklists in which case study methodology either does not feature (and so almost inevitably becomes lost) or in which it is misinterpreted and misunderstood. There is scope for developing guidance and publication standards to help those reporting, publishing and using evidence from case studies.

## Supplementary Information

**Additional file 1.**

## Data Availability

Data sharing is not applicable to this article as no datasets were generated or analysed during the current study.

## Abbreviations

MRC: Medical Research Council
RCT: Randomised Controlled Trial
