# Supplementary material for: Evaluating complex interventions in context: systematic, meta-narrative review of case study approaches
Source: BMC Med Res Methodol. 2021 Oct 25;21:225. doi: 10.1186/s12874-021-01418-3 (PMC8543916; doi:10.1186/s12874-021-01418-3)
Supplement: Supplementary file 1 — Additional file 1. [file 12874_2021_1418_MOESM1_ESM.pdf]

APPENDIX 1 – SUMMARY OF SEARCH RESULTS (CYCLE 2)

**Database**	**Interface**	**Coverage**	**Date**	**Hits**
Medline (Ovid MEDLINE® Epub Ahead of Print, In-Process & Other Non-Indexed Citations, Ovid MEDLINE® Daily and Ovid MEDLINE®) 1946 to present	OvidSP	1946-present	22/11/2019	2175
Embase	OvidSP	1974-present	22/11/2019	1032
PsycINFO	OvidSP	1806-present	22/11/2019	783
CAB Abstracts	OvidSP	1973 to 2019 Week 36	22/11/2019	190
Science Citation Index, Social Sciences Citation Index and Arts & Humanities Citation Index	Web of Science Core Collection	1945-present	22/11/2019	1083
ERIC	EBSCOHost	1966-present	22/11/2019	78
CINAHL	EBSCOHost	1982-present	22/11/2019	1002
ASSIA	Proquest	1987-present	22/11/2019	13
Sociological Abstracts	Proquest	1952-present	22/11/2019	10
PAIS Index	Proquest	1914-present	22/11/2019	10
**Total:**				**6376**
Duplicates removed:				2151
**Final Total:**				**4225**
